# Microplastics amplify the pro-inflammatory response to fungal mycelial fragments and spores in neutrophil-like cells

**DOI:** 10.3389/ftox.2026.1718466

**Published:** 2026-02-10

**Authors:** Anani K. Afanou, Andreas Solberg Sagen, Francesco Barbero, Ilaria Zanoni, Anna Costa, Øyvind P. Haugen, Shan Zienolddiny-Narui

**Affiliations:** 1 STAMI, National Institute of Occupational Health, Oslo, Norway; 2 UIO, Institute of Oral Biology, University of Oslo, Oslo, Norway; 3 UNITO, Department of Chemistry, University of Torino, Torino, Italy; 4 CNR-ISSMC, Institute of Science, Technology and Sustainability for Ceramics, National Research Council of Italy, Faenza, Italy

**Keywords:** fungal particles, high-density polyethylene microplastics, immune responses, mixed particle exposure, Toll-like receptor activation

## Abstract

**Background:**

Microplastic pollution has emerged as a global environmental crisis with potential adverse consequences on human health. Mixtures of microplastics with fungal particles including mycelial fragments or spores are highly probable exposure scenarios occurring in various occupational settings or in moldy built indoor environments. However, immunotoxic outcomes associated with such exposure remain poorly characterized. Most studies have focused on single‐exposure components. Here, we investigated, for the first time, the immunotoxic effects of microplastics mixed with spores or mycelial fragments from *Aspergillus fumigatus* on human neutrophil-like cells.

**Materials and methods:**

Differentiated HL60 neutrophil-like cells were exposed to 0–100 μg/mL HDPE microplastics mixed with 10^6^ heat-inactivated mycelial fragments or spores for 24 h.

**Results and discussion:**

HDPE combined with fungal fragments induced significant release of IL‐6 and IL‐8 while the mixtures with fungal spores induced only IL‐6 release from the neutrophil-like cells. Most importantly, we observed a trend of decreasing IL‐6 levels with increasing doses of HDPE microplastics in mixture with fungal particles, indicating possible dysregulation of the pro-inflammatory response. The tested doses of HDPE microplastics in mixture with fungal particles showed no significant acute effects on the cell viability. Using HEK293‐TLR reporter cells, we found no significant activation of TLR2 and TLR4 by HDPE microplastics, fungal particles, or their combination, suggesting that the release of IL‐6 and IL‐8 is induced through other innate immune-signaling pathways. Taken together, fungal particles as microbial contaminants, seem to be the main drivers of the immune responses triggered by exposure to mixed HDPE microplastics and fungal particles. Among these, fungal mycelial fragments appear to be the most potent compared to fungal spores that are typically monitored for risk assessments.

## Graphical Abstract

**Figure:**
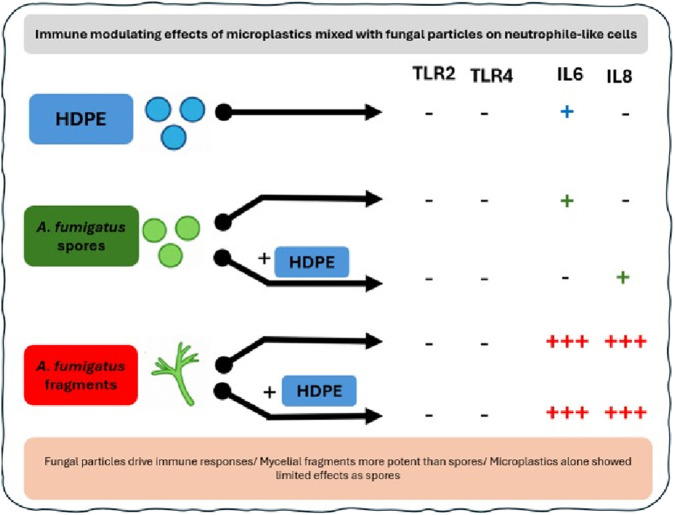

## Highlights

- Mixtures of microplastics with fungal fragments induced stronger and more complex pro-inflammatory responses than mixtures with fungal spores.
- Increasing doses of microplastics seem to reduce the levels of released IL-6.
- The tested doses had no acute effect on cell viability.
- The tested doses induced no TLR2 and TLR4 activation through the NF-κB pathway, indicating the involvement of other pathways leading to IL-6 or IL-8 release

## 1 Introduction

Plastic production, use, and improper plastic waste management constitute major drivers of the emerging planetary pollution crisis. Plastic degradation releases micro- and nanoplastics (MNPs) that infiltrate and bioaccumulate across diverse biomes, including human tissues. Although health impact assessments remain in preliminary stages, human biomonitoring studies revealed the presence of MNPs in feces (Yan et al., 2022), placenta (Ragusa et al., 2022), blood (Leslie et al., 2022), lung tissues (Jenner et al., 2022), testes (Hu et al., 2024), and brain tissues (Amato-Lourenço et al., 2024; Nihart et al., 2025). Epidemiological evidence links MNP exposure to a 4.5-fold increased risk of myocardial and cerebral infarcts and mortality (Marfella et al., 2024). Studies have been shown that microorganisms and other contaminants can effectively adsorb onto MNPs (Joo et al., 2021; McCormick et al., 2014) that function as vectors, contributing to the spread of pathogenic, allergenic, and antigenic microbial components. In this regard, available data are limited to bacterial biofilm formation, while less attention has been devoted to such interaction with fungal particles (Gkoutselis et al., 2021).

High-density polyethylene (HDPE) represents a significant source of microplastics due to its widespread application in packaging, construction, and household products, with degradation and fragmentation occurring through mechanical use and environmental weathering (Chinglenthoiba et al., 2025). The persistent environmental presence of HDPE microplastics and their cross-species health implications have emerged as a critical research domain (Naz et al., 2024; Polenogova et al., 2025). The primary concerns are the immunotoxicity and immunomodulatory effects caused by HDPE microplastics (MPs), which have been identified as one of the predominant polymer types contributing to environmental contamination (Beijer et al., 2022; Polenogova et al., 2025). Despite documented immune alterations across fish, invertebrate, and mammalian models, mechanistic understanding at molecular and cellular levels remains limited (Huang et al., 2023; Islam et al., 2024; Li et al., 2024). Moreover, conflicting findings exist on whether HDPE MPs suppress or activate immune responses, with some studies reporting immune suppression and others indicating pro-inflammatory activation (Park et al., 2024; Polenogova et al., 2025; Wolff et al., 2023). Additional uncertainty surrounds the modulatory role of particle size, weathering-induced surface modifications, and co-transported contaminants. This knowledge gap limits both exposure risk assessment and intervention development as immune dysfunction can lead to increased susceptibility to pathogens and chronic inflammation (Xu et al., 2024; Yang et al., 2023).

Both MPs and fungal components independently induce adverse cellular and systemic responses, including oxidative stress, inflammation, genotoxicity, and immune dysregulation. Co-exposure amplifies these concerns as microplastics act as vectors that enhance fungal pathogen and mycotoxin transport, cellular uptake, and bioavailability, potentially altering their pathogenic mechanisms (Gkoutselis et al., 2024; Gkoutselis et al., 2021; Klauer et al., 2024; Zhao et al., 2024). Since available data are limited to pristine polystyrene test materials, the specific molecular mechanisms induced by environmentally relevant microplastics and the health-associated hazards remain unknown (Jiang et al., 2020; Mitrano and Wohlleben, 2020; Prata et al., 2020). In contrast, the molecular key events induced by fungal exposure and related disease outcomes are well characterized. For example, tissue damage through oxidative stress and inflammatory responses upon exposure to fungal spores, mycotoxins, and volatile organic compounds has been reported (Holme et al., 2020). *Aspergillus spp.* with *Aspergillus fumigatus*, in particular, are among the most frequently studied fungal species as their spores and fragments are highly prevalent in various indoor and occupational settings (Li et al., 2022; Mousavi et al., 2016). *A. fumigatus* is also a well-studied pathogenic fungus, often associated with fungal infection and negative health outcomes in subjects with altered immune systems (Latgé, 2001; Latgé and Chamilos, 2019). Spores from *A. fumigatus* have been measured in working environments, such as industrial composting and waste treatment facilities (Kontro et al., 2022; Schlosser et al., 2016). Inhaled fungal particles can easily reach the alveoli where they interact with alveolar epithelial cells and macrophages through pathogen recognition receptors (PRRs), initiating inflammatory cascade reactions with the subsequent release of chemokines and cytokines (Adler et al., 1994; Allermann and Poulsen, 2000; Devalia, 1993). The chemokine IL-8 is known as a neutrophilic chemotactic factor as it induces the recruitment of neutrophils and other granulocytes to the particle deposition site (Kolaczkowska and Kubes, 2013). Sustained neutrophilic/granulocyte accumulation in the lungs is associated with an oxidative burst that, during chronic exposure, leads to progressive tissue damage (Johansson and Kirsebom, 2021; McGovern et al., 2016).

Given neutrophils’ central role in innate immunity and their numerical predominance (55%–60%) among circulating leucocytes, characterizing the effects of particulate matter on these cells represents a critical research priority (Johansson and Kirsebom, 2021). Their infiltration into the lungs, as hallmark of inflammatory responses, following airway exposure to polyethylene (PE) and polypropylene (PP) microplastics has been documented in rodents (Jung et al., 2025; Tomonaga et al., 2024). Similarly, *A*. *fumigatus* spore exposure induces neutrophil migration in Transwell-based *in vitro* lung models (Feldman et al., 2020). However, neutrophil responses to co-exposure scenarios involving microplastics and fungal particles remain unexplored. Many *in vitro* studies have used granulocyte-like differentiated human promyelocytic leukemia HL-60 cells as a surrogate for human granulocytes (Blanter et al., 2021; Timm et al., 2009). These cells possess key neutrophil functions, including pathogen and exogenous particle detection via PRRs, particularly Toll-like receptors (TLRs) that recognize fungal cell wall components (Hayashi et al., 2003; Sato et al., 2003; Shuto et al., 2007).

The complex physicochemical properties of microplastics, particularly their ability to simultaneously bind organic compounds and contaminants, demand a holistic assessment approach for potential human health risk evaluation (Galloway et al., 2017; Ramsperger et al., 2020). Current toxicological research has predominantly focused on the isolated effects of individual polymers or single contaminants, often overlooking the prevailing real-world scenarios involving complex environmental mixtures. This reductionist approach likely underestimates health risks as interactions between different pollutants can lead to synergistic or antagonistic effects not predictable from single-exposure studies. There is paucity of immunotoxicity data on MPs in combination with microbial contaminants, particularly from fungi (Hirt and Body-Malapel, 2020; Prata et al., 2020). This knowledge gap is critical given that airborne particulate matter in both indoor and outdoor environments consistently contains microplastics alongside fungal particles (Dris et al., 2017; Zhai et al., 2018), representing the actual human exposure profile.

To explore the neutrophilic immunotoxic effects associated with the complex mixture of microplastic and fungal particles, we exposed neutrophil cells differentiated from HL-60 cells to HDPE particles mixed with spores or mycelial fragments from *A. fumigatus*. We evaluated the toxic effects on cell viability and the cellular pro-inflammatory responses at protein levels. Moreover, we assessed the NF-κB-induced response through the activation of TLR2 and TLR4 in reporter cells.

## 2 Materials and methods

### 2.1 Test particles

High-density polyethylene (HDPE) particles (with size characteristics using DLS: number-based median hydrodynamic size, D_n_50 = 2.7 µm; 10th percentile hydrodynamic size, D_n_10 = 1.3 µm; 90th percentile hydrodynamic size, D_n_90 = 5.8 µm) were provided by the University of Torino (UNITO), under the PlasticsFatE consortium, as HDPE particles suspended in sterile endotoxin-free water (Alfa Aesar #J-65589-K2) containing 0.025% BSA. For the fungal particle, *A*. *fumigatus* 1863 (strain A1258 FGSC) from the Fungal Genetics Stock Center (University of Missouri, Kansas City, United States) was used. Fungal spores (AFS) and mycelial fragments (AFM) were prepared as previously described by Afanou et al. (2014). The fungal spores and fragments were heat-inactivated at 90 °C for 40 min following the protocol from Beisswenger et al. (2012).

The concentrations of the test particles (HDPE, AFM, and AFS) were measured using gravimetry and microscopy. For the gravimetric analysis, 100 µL of the diluted stock suspensions of HDPE, AFM, and AFS was transferred dropwise onto conditioned and pre-weighted polycarbonate membrane filters (0.4-µm pores, Millipore #HTTP02500 Merck Darmstadt, Germany) and were then dried for 4 h under sterile conditions. The filters were then conditioned for 24 h, before being weighed using a high-precision scale (Sartorius AG, MC210, Göttingen, Germany). The dry weight of the particles was determined as the average of three replicates, adjusted using control measurements of environmental changes.

A preliminary light microscopy analysis of the fungal particles was performed prior to field emission scanning electron microscopy (FESEM) analysis. In brief, 10 µL of the diluted stock suspensions of AFM and AFS was applied to a hemocytometer, and the spores and fragments were counted using a light microscope (Discover Echo, Revolve, San Diego, United States). The mean concentrations were estimated from particles counted in 10 fields of the hematocytometer.

To prepare the fungal and HDPE particle mixture, 10 µL of AFM or AFS stock suspensions (10^10^ particles/mL) was added to 10 mL of HDPE at 10, 100, and 1,000 μg/mL in endotoxin-free water (Sigma-Aldrich #A8806 Merck Darmstadt, Germany) containing 0.025% BSA. Additionally, controls were prepared with an equivalent concentration of AFM or AFS in 0.025% BSA. All suspensions were mixed with 200 rpm orbital shaking for 24 h at room temperature (RT).

### 2.2 FESEM analysis of the particles

FESEM analysis was performed to assess size characterization and distribution of the test materials. In brief, 100 µL of the HDPE and AFM/AFS suspension mixtures was filtered through 0.4-µm pore polycarbonate filters using a membrane vacuum filtration system (Advantec #311220 Mikrolab Frisenette, Danmark). Each membrane was dried for 2 h under sterile conditions, before being transferred to a two-sided adhesive tab mounted onto an aluminum stub. The samples were then coated with 5 nm platinum in a Cressington super coating machine (Cressington Scientific Instruments Ltd., Watford, United Kingdom). Coated samples were visualized using an FESEM (Hitachi High-Tech, SU6600 Analytical FESEM, Tokyo, Japan). The microscope was operated in the secondary electron imaging mode, with an acceleration voltage of 15 keV, an extraction voltage of 1.8 kV, and a working distance of 10–11 mm. Particle numbers were assessed in randomly selected fields to achieve a total of 200 particles. For the particle size characterization, Esprit feature analysis (Quantax EDS for SEM, Bruker) was performed in back-scattered imaging mode, focusing on particle length, width, average diameter, and equivalent diameters in randomly selected FESEM fields. Assuming a homogenic dispersion of particles over the membrane, the number of particles was determined following the method described by Eduard and Aalen (1988). The number of particles was estimated using the following formula:

$$C=\frac{N\times Afilter\times D}{K\times Asem},$$ (1)

where C is the number of particle concentration, *N* is the number of counted particles, *A*_*filter*_ is the filter area (in mm^2^), *K* is the number of SEM areas, and *A*_*SEM*_ is the imaging area (in mm^2^) in SEM.

### 2.3 HL-60 cell culture and differentiation

The human promyelocytic cell line HL-60 (ATCC #CCL-240 LGC, Lancashire, United Kingdom) was cultured in Roswell Park Memorial Institute 1640 (RPMI; Gibco #61870 Thermofisher Scientific, Waltham MA, United States) medium supplemented with 10% heat-inactivated endotoxin-free FBS. The cells were sub-cultured every 2–3 days at 37 °C in a 5% saturated CO_2_ atmosphere with 95% relative humidity. The cells were differentiated to mature granulocytes (dHL-60 cells) using 1.25% dimethyl sulfoxide (DMSO; MP Biosciences #196055 Irvine, CA, United States) over 5 days as previously described in the literature (Manda-Handzlik et al., 2018; Sham et al., 1995). dHL-60 cells were harvested by centrifugation at 200 RCF for 5 min and resuspended in RPMI with 10% FBS. The cell concentration was determined using an automated cell counter (ChemoMetec, NucleoCounter NC-200, Gydevang, Denmark) prior to seeding for various exposure treatments.

### 2.4 Toll-like receptor activation

To evaluate NF-κB activation via TLR2 and TLR4, we used HEK293 reporter cell lines engineered to express TLR2 or TLR4 and produce secreted alkaline phosphatase (SEAP) upon pathway stimulation. In brief, TLR2 (InvivoGen #hkb-htlr2 Toulouse, France) and TLR4 (InvivoGen #hkb-htlr4) HEK-Blue reporter cells were grown in Dulbecco’s modified Eagle’s medium (DMEM; Gibco #31966) supplemented with 10% heat-inactivated endotoxin-free fetal bovine serum (FBS; Biowest #S1860), 100 U/mL penicillin (Biowest #L0022 Nuaillé, France), 100 μg/mL streptomycin (Biowest #L0022), 100 μg/mL normocin (InvivoGen #ant-zn), and 1xHEK Selection-Blue (InvivoGen #hb-sel).

The exposure to particle suspensions was performed following the method previously described by Brummelman et al. (2015). In brief, 180 µL of HEK-Blue hTLR2 or HEK-Blue hTLR4 cells (2.8 × 10^4^ cells/mL) was seeded in a 96-well plate with each condition tested in technical triplicate. Considering the buoyancy properties of the HDPE particles, additional experiments using inverted 12-well inserts were performed. As such, we believed that there was better interaction between floating HDPE particles and the cells grown on the basolateral side of the insert. A detailed description of the inverted insert system is available in the Supplementary Material. Following 24 h of incubation to allow cell attachment, cultures were treated with 20 µL of 10× concentrated particle suspensions or endotoxin-free water (vehicle control), yielding final 1× particle concentrations in a total volume of 200 µL. As positive controls, lipoteichoic acid (LTA, 100 ng/mL) and lipopolysaccharide (LPS, 100 ng/mL) were used to stimulate TLR2 and TLR4, respectively. After 24 h of exposure, receptor activation was quantified using the QUANTI-Blue colorimetric assay (InvivoGen #rep-qbs). The supernatant of each treatment (20 µL) was transferred to a new 96-well plate containing 180 µL of QUANTI-Blue reagent. The plates were incubated for 3 h at 37 °C in humidified 5% CO_2_ atmosphere, and the absorbance was measured at 649 nm using a microplate reader (Agilent Technologies, BioTek Synergy Neo2, Santa Clara, United States). The absorbance readings were blank-adjusted before being normalized to the average of negative controls. Final data from the HEK293-TLR reporter cells were based on two independent experiments with three replicates of each treatment.

### 2.5 Cell viability by Alamar Blue assay

In brief, 100 µL of dHL-60 cells (5 × 10^4^ cells/mL or 5,000 cells/well) was seeded in phenol-free RPMI medium (Gibco #11835) supplemented with 10% FBS in 96-well plates; 15 μL of 10× concentrated particle suspensions and controls was diluted with 35 µL of culture medium, added to each well in technical triplicates, and incubated for 24 h.

Subsequently, 50 µL of 40% Alamar Blue solution (Invitrogen #DAL1100 Thermofisher Scientific, Waltham MA, United States) in culture medium was added to each well and incubated for 3 h. The plate was then centrifuged at 300 RCF for 5 min, and 100 µL supernatant was transferred to a black-walled 96-well plate (Nunc, Roskilde, Denmark). The fluorescence of the cell-free solution was measured using a plate reader at 590 nm emission and 560 nm excitation. The sample readings were blank-adjusted before being normalized to the average of the reference control. This assay was repeated in three independent experiments.

### 2.6 Assessment of pro-inflammatory markers (IL-6 and IL-8) by ELISA

To quantify interleukin-6 (IL-6) and interleukin-8 (IL-8) levels, selected as pro-inflammatory markers, dHL-60 cells were seeded in 6-well plates (Sarstedt, Nümbrecht, Germany) at 1.2 × 10^6^ cells/well in 1.8 mL complete culture medium and incubated for 15 min prior to the addition of treatment compounds. Then 200 μL of treatment was added to each well in technical replicates and incubated for 24 h. The supernatant was then collected by centrifugation at 300 RCF for 5 min to remove cells and debris and stored at −80 °C prior to analysis. The exposure and subsequent supernatant were collected from three independent experiments. Analysis of the inflammatory markers was performed using ELISA kits from PeproTech (PeproTech #900-K16 for IL-6 and PeproTech #900-K18 for IL-8, Cranbury, NJ, United States). The ELISA kits were used following the manufacturer’s instructions. In brief, 96-well plates were coated with 100 µL of the capture biotinylated antibody at 1 μg/mL in PBS and incubated overnight at room temperature (RT). The plates were then washed in a microplate washer four times followed by 5 min of incubation with 300 µL of washing buffer (PeproTech #900-K00). The wells were blocked with 100 µL of blocking buffer for 1 h at RT followed by washing as previously described. A measure of 100 µL of the cell supernatant and standards was transferred to the coated microplates and incubated for 2 h at RT. The samples and standards were discarded, and the wells were washed as described previously. Following this, 100 µL of avidin–HRP-conjugated detection antibody (at 1:2000 dilution) was added to the well and incubated for 2 h at RT. The plates were washed again, and 100 µL of substrates was added to the well followed by 30 min of incubation at RT for color development. The absorbance was monitored every 5 min at 405 nm with wavelength correction at 650 nm in Synergy Neo 2 Microplate reader GEN 5 software (BioTek Instruments, United States). All samples were analyzed in triplicate, and final levels of pro-inflammatory markers were estimated using the four-parameter (4-PL) regression (Dotmatics, GraphPad Prism 9, Boston, United States).

### 2.7 Data and statistical analysis

Data were organized as metadata with experimental replicates using Microsoft Excel, and statistical analysis was performed in Stata 18.5 (StataCorp LP, College Station, TX) using the non-parametric Dunn test package for group comparisons followed by false discovery rate (FDR) adjustment (Benjamini–Hochberg method with a significant p-value <0.05). The non-parametric test was used because of the small sample size and the violation of the normality assumption.

## 3 Results

### 3.1 Material characteristics

**FIGURE 1:**
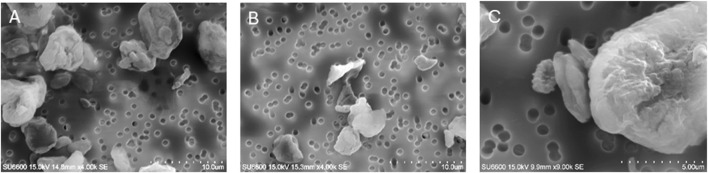
Field emission scanning electron microscopy (FESEM) micrographs of HDPE microplastics **(A)**, along with the mixtures of HDPE MPs and *A. fumigatus* fragments **(B)** and spores **(C)**.

High-resolution micrographs of HDPE microplastics, fungal fragments, and the mixtures are presented in Figure 1. Further characterization of the particle size distribution using FESEM–EDS with Esprit feature analysis of the test materials is summarized in Table 1.

**TABLE 1:** Average mass per particle by gravimetry and size characteristics and distribution using FESEM.

Test materials	Size characteristic	10th percentile	50th percentile	90th percentile	N particles
AFS (0.18 pg/particle)^a^	Length (µm)	1.77	2.99	6.13	346
	Width (µm)	1.22	1.96	3.71	
	Average diameter (µm)	1.58	2.55	5.18	
	Equivalent diameter (µm)	1.44	2.3	4.33	
	Aspect ratio	1.24	1.43	1.88	
AFM (1.60 pg/particle)^a^	Length (µm)	4.41	6.62	11.33	643
	Width (µm)	3.06	4.29	6.52	
	Average diameter (µm)	3.91	5.68	9.27	
	Equivalent diameter (µm)	1.54	3.22	5.97	
	Aspect ratio	1.28	1.54	2.05	
HDPE (0.05 pg/particle)^a^	Length (µm)	1.97	4.13	8.36	834
	Width (µm)	1.22	2.81	5.23	
	Average diameter (µm)	1.71	3.6	7.05	
	Equivalent diameter (µm)	1.54	3.22	5.97	
	Aspect ratio	1.23	1.49	2.03	
AFS–HDPE	Length (µm)	2.08	4.01	8.24	625
	Width (µm)	1.39	2.69	5.27	
	Average diameter (µm)	1.8	3.45	6.89	
	Equivalent diameter (µm)	1.6	2.99	5.84	
	Aspect ratio	1.25	1.49	2.00	
AFM–HDPE	Length (µm)	2.31	5.1	10.69	458
	Width (µm)	1.65	3.43	6.55	
	Average diameter (µm)	2.00	4.46	8.84	
	Equivalent diameter (µm)	1.79	4.01	7.54	
	Aspect ratio	1.22	1.44	1.93	

^a^ Average mass of the particles determined using gravimetry.

### 3.2 Cell viability of dHL-60 cells by Alamar Blue assay

**FIGURE 2:**
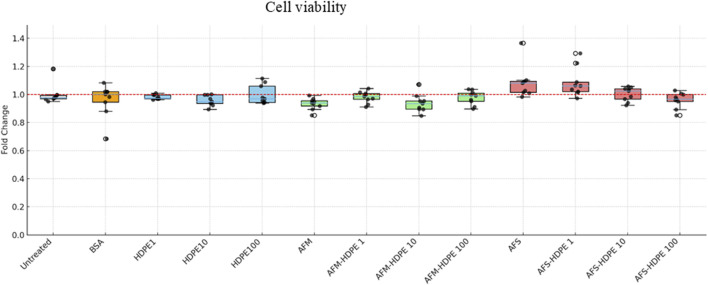
Box plots showing relative changes in reduced resazurin as an indicator of cell viability. Differentiated HL-60 cells were treated with HDPE (1, 10, or –100 μg/mL), or 10^6^ AFM/AFS particles per mL with varying concentrations of HDPE (with HDPE 1 = 1 μg/mL; HDPE 10 = 10 μg/mL; HDPE 100 = 100 μg/mL). Each box represents data from three independent experiments with three technical replicates.

The effect of the test materials on cell viability is summarized as box plots in Figure 2. The results reported here are based on changes in the reduction of resazurin following cell exposure to the test materials. All data were adjusted for background signals and normalized to the negative control. Overall, no significant effects on cell viability were detected. However, a slight reduction in cell viability, although not significant, was observed with cells exposed to 10^6^ AFM per mL, mixture of 10 μg/mL HDPE + 10^6^ AFM per mL, and mixture of 100 μg/mL HDPE +10^6^ AFS per mL. Of note, a slight reduction (but not significant) in cell viability was observed in cells exposed to increasing concentrations of HDPE in a mixture with fungal spores.

### 3.3 Toll-like receptor activation by HEK293 reporter cell assay

**FIGURE 3:**
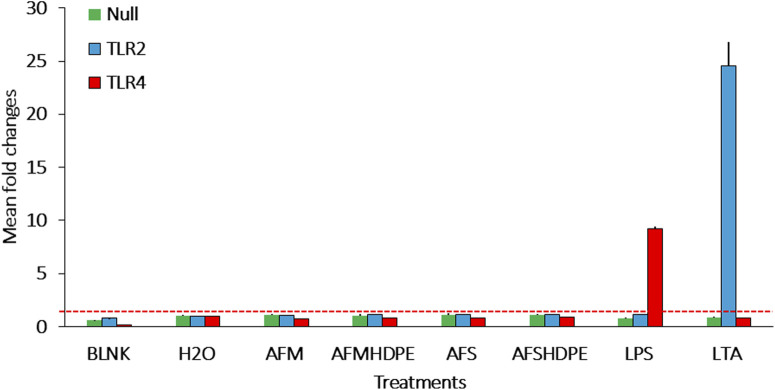
Bar plots of the fold changes following submerged exposure of reporter cells to test materials that include AFMHDPE (AFM (10^6^/mL) + HDPE (100 μg/mL)), AFM (10^6^/mL), AFSHDPE [AFS (10^6^/mL) + HDPE (100 μg/mL)], AFS (10^6^/mL) particles, H_2_O as the negative control, and positive controls [LPS (100 ng/mL) for TLR4 and LTA (100 ng/mL) for TLR2]. The blank control served as the background response without cells. The red stippled line indicates fold change level 2, which was considered the activation threshold. Data from two independent experiments are shown.

Figure 3 shows the fold change in SEAP activity after 24-h exposure of reporter cells to 10^6^ AFM/mL or 10^6^ AFS/mL and the mixtures of 10^6^ AFS/mL + HDPE (100 μg/mL) and 10^6^ AFM/mL + HDPE (100 μg/mL). The fold change was calculated by dividing the absorbance of test materials by the average of controls across two independent experiments. Following 24 h of exposure, neither TLR2 nor TLR4 was activated by any of the test materials. However, the positive controls for TLR2 and TLR4 induced significant activation of the respective TLRs. Similarly, data from the inverted insert exposure model revealed that HDPE particles do not significantly activate the two receptors (Supplementary Figure S1).

### 3.4 Measured pro-inflammatory markers by enzyme-linked immunosorbent assay

**FIGURE 4:**
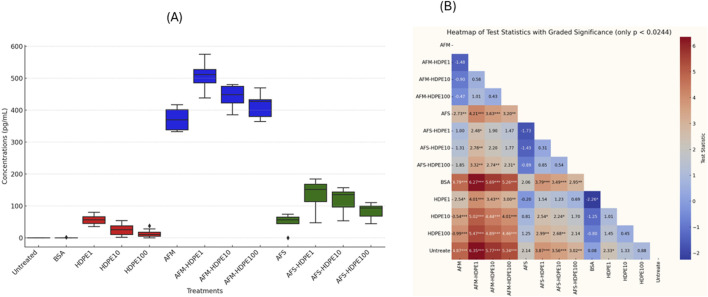
Box plots of IL-6 concentrations released **(A)** by the cells after 24 h of exposure to test materials and controls with **(B)** heatmap of test statistics. Cells were exposed to either HDPE particles alone (1–100 μg/mL) or 10^6^ AFM/AFS particles per mL mixed with varying concentrations of HDPE (1–100 μg/mL). Comparison results of treatments using Dunn test statistics with reported FDR-adjusted p-values provided as heatmap **(B)**. Data analysis and visualization are based on three independent experiments. (*) p-value <0.024; (**) p-value <0.01; (***) p-value <0.001.

**FIGURE 5:**
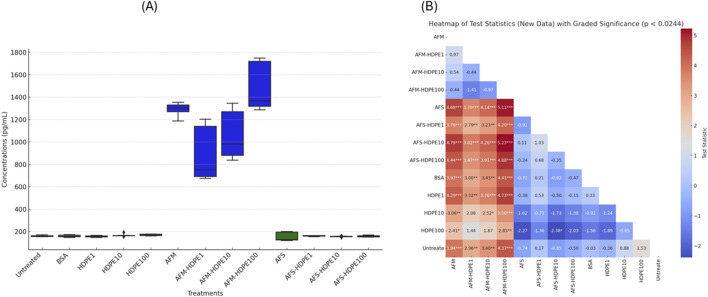
Box plots of IL-8 concentrations released **(A)** by the cells after 24 h of exposure to test materials and controls with **(B)** heatmap of test statistics. Cells were exposed to either HDPE particles alone (1–100 μg/mL) or 10^6^ AFM/AFS particles per mL mixed with varying concentrations of HDPE (1–100 μg/mL). Comparison results of treatments using Dunn test statistics with reported FDR-adjusted p-values provided as heatmap **(B)**. Data visualization and analysis are based on three independent experiments. (*) p-value <0.024; (**) p-value <0.01; (***) p-value <0.001.

Figures 4, 5 show the levels of IL-6 and IL-8 measured by ELISA in the cell medium after 24 h of exposure with dHL-60. No detectable levels of IL-6 were measured in the negative (H_2_O) control-treated and vehicle (BSA) control-treated cells, while levels above the detection limit were measured in the HDPE, AFM, AFS, and mixture treatments. Markedly higher levels of IL-6 and IL-8 were measured in the HDPE mixtures with AFM. With AFS mixtures and HDPE, only IL-6 was significantly released. Importantly, all the mixtures of HDPE with fungal fragments induced higher levels of IL-6 and IL-8 than HDPE alone, while AFS mixture induced only IL-8. Considering the pro-inflammatory potential of the test materials, AFM and AFM mixtures induced the highest levels of IL-6 and IL-8, followed by AFS mixtures and HDPE alone with the lowest response.

No significant levels of IL-6 were particularly detected with cells exposed to the vehicle control (0.0025% BSA) compared to the untreated cell control, but considerably higher levels were measured with cells exposed to 1 μg/mL HDPE. We also observed a decreasing level of IL-6 in response to increasing doses of HDPE, with the highest levels at 1 μg/mL and the lowest at 100 μg/mL. Significantly higher levels of IL-6 were induced by 1 μg/mL (but not at 10 and 100 μg/mL) HDPE alone compared to the untreated cells. IL-6 secretion was also significantly higher in cells treated with the mixtures of AFS + HDPE (1–100 μg/mL) compared to the untreated cells and vehicle controls, but the levels with AFS alone did not differ from those with the controls. Another important observation is that increasing HDPE concentrations (1–100 μg/mL) alone or in mixture with 10^6^ AFM/mL or 10^6^ AFS/mL induced decreasing levels of IL-6 concentrations.

For IL-8, approximately 160 ng/mL was measured in the untreated cell control, and this level was similar to the levels released by cells treated with vehicle control (0.0025% BSA), HDPE, or AFS alone. Moreover, cells exposed to 10^6^ AFM/mL alone or in mixture with HDPE (1–100 μg/mL) released over 5× more the mean levels of IL-8 compared to the controls, HDPE, or AFS alone. The differences in the IL-8 levels released by cells treated with 10^6^ AFM/mL and controls or HDPE alone (1–100 μg/mL) were statistically significant, but not between AFM alone and AFM mixed with HDPE (1–100 μg/mL). The increasing level of HDPE in the mixture with AFM is positively correlated with IL-8 release.

## 4 Discussion

In the present study, we evaluated the immunotoxicological effects of HDPE microplastics in combination with *A. fumigatus* particles (spores and fragments) on neutrophil functions using differentiated HL-60 cells as an *in vitro* model. Cells were exposed to concentration-dependent HDPE microplastics (1–100 μg/mL) co-administrated with a constant concentration (10^6^ particles/mL) of *A*. *fumigatus* conidia (AFS) or mycelial fragments (AFM).

Cytotoxicity assessment revealed no significant acute effects on the cell viability across the tested concentration range. Similarly, pattern recognition receptor activation analysis demonstrated no significant upregulation of TLR2 and TLR4 expression following particle exposure. However, differential pro-inflammatory responses with IL-6 and IL-8 were observed, indicating particle-specific immunomodulatory effects. The secretion of IL-6 showed a bimodal dose–response relationship in AFM–HDPE co-exposures, with peak release at the lowest HDPE concentrations (1 μg/mL + 10^6^ AFM/mL) and significant attenuation at the highest concentration (100 μg/mL HDPE + 10^6^ AFM/mL). In contrast, IL-8 release demonstrated a positive dose-dependent relationship with increasing HDPE concentrations (1–100 μg/mL) exclusively in the presence of AFM particles. Although not statistically significant, the addition of HDPE at two lower doses (1 and 10 μg/mL) seemed to induce a reduction in IL-8 levels relative to AFM alone. This suggests a two-phase effect such as hormesis responses with the HDPE microplastics; however, further experimental studies are required for any consistent conclusion. Notably, HDPE–conidia mixtures did not elicit considerable IL-8 responses, suggesting morphotype-specific inflammatory potential.

To the best of our knowledge, this is the first study to reveal such differential effects of fungal particle morphotypes in the presence of HDPE microplastics. Our findings suggest additive inflammatory effects between HDPE microplastics and *A*. *fumigatus* mycelial fragments, as evidenced by enhanced IL-8 release in co-exposure scenarios compared to individual particle treatments. These results highlight the importance of considering particle morphology and co-contaminant interactions in environmental health risk assessments.

None of the tested materials and combinations negatively affected the neutrophil-like cell viability. The tested doses of HDPE particles alone or in mixture with fungal particles revealed no significant alteration of the neutrophil-like cell viability after 24 h of exposure. This result can be explained by the relatively low numbers of fungal particles per neutrophil cells (three fungal particles per cell). In fact, 15 µL of 10^6^ particles/mL was used per well, which is equivalent to 15,000 particles per 5,000 cells per well or 3 particles per cell. Similar results were obtained with even much higher fungal particle concentrations (spores and fragments from *A. fumigatus*) with THP1 macrophages (1,400 particles per cell) (Øya et al., 2018) and in mature human macrophages exposed to HDPE particles (Xing et al., 2002). However, contradictory results were found by Herrala et al. (2023), who exposed gastrointestinal cells to micro-sized PE particles and ethanol-extracted compounds from the particles. Their results revealed that the micro-sized PE particles and the ethanol-extracted compounds reduced cell viability and increased oxidative stress when intestinal cells were exposed to high concentrations (0.25–1 mg/mL) of test materials. The relatively higher doses (>200 μg/mL) used by Herrala et al. may explain such discrepancy as they only observed decreasing cell viability from 500 μg/mL. Although cytotoxicity is the basic toxicity parameter commonly used to evaluate potential hazards of any suspected component, most of the particulate matter pollutants, at environmentally relevant exposure doses, do not cause acute cell death or alter cell viability *in vitro*. However, these exposure agents can induce many immune responses that, if not regulated back to homeostasis, can lead to diseases.

As summarized in Figure 3, co-exposure of HEK293 reporter cells to 10^6^ fungal particles/mL and 100 μg/mL HDPE microplastics did not activate TLR2 or TLR4. This result is in contradiction with previously reported data on *A. fumigatus*, revealing significant TLR2- and TLR4-dependent immune responses in macrophages (Meier et al., 2003). This discrepancy may be attributed to two factors. First, the doses applied in the present study are six times lower than those used by Meier et al. We consider the dose of fungal particles tested in our study as a more environmentally relevant exposure dose (Eduard, 2009). The second factor is heat inactivation applied to the fungal materials, which was implemented as a safety measure during the handling of *A. fumigatus* particles as well as to prevent invasion of the cell culture medium. As fungal walls are adorned with a variety of proteins that can act as ligands for immune receptors, including TLRs, heat treatment may significantly alter the conformation of such protein ligands, in the way that their affinity to receptors of the immune system was lost (Ibe and Munro, 2021). In fact, thermal denaturation can significantly impact the three-dimensional structure of protein ligands, which are crucial for their recognition by immune receptors, such as TLRs (Van De Veerdonk et al., 2008).

From the cells exposed to HDPE–AFM, the secretion of IL-8 markedly increased compared to the negative control, HDPE microplastics, and AFS treatments. This can be attributed to the *A*. *fumigatus* spores’ ability to evade the immune system due to the hydrophobin protecting layer, which is absent in hyphal structures. The exposed mycelium is not protected from detection by PRRs (Garcia-Rubio et al., 2020) and thus capable of inducing pro-inflammatory responses (Øya et al., 2018). Data similar to our findings on IL-8 secretion were reported with polymorphonuclear (PMN) cells exposed to fungal particles (Braedel et al., 2004). Although not investigated in the present study, it is likely that IL-8 secretion may be initiated through dectin-1 receptor activation (Meier et al., 2003; Werner et al., 2009), but not through TLR2 and TLR4, as previously reported (Meier et al., 2003). It is well established that binding to TLRs mediates expression and secretion of cytokines in sentinel cells (El-Zayat et al., 2019). It is also known that *A. fumigatus* antigens activate TLR2 and TLR4, but it remains unclear whether MNPs interact with similar receptors in immune cells (Blackburn and Green, 2022; Braedel et al., 2004).

TLRs are one of the most important receptor families for detection of pathogens and foreign objects among sentinel cells (Seitz, 2003). Neutrophils and the dHL-60 neutrophilic cell model are both reported to express TLR2 and TLR4 (Saegusa et al., 2009; Shuto et al., 2007). In particular, TLR2 and TLR4 are important receptors for detection of cell wall microbial components, such as LPS and LTA from bacteria, along with chitin and zymosan from fungi. LTA (from bacteria) and zymosan (from fungi) can induce immune responses through TLR2 activation (Ikeda et al., 2008), while LPS from bacteria binds to TLR4. As summarized in Figure 3, neither AFM nor AFS bound to any of these receptors. In the case of AFS, this was expected as undamaged and non-germinated *A. fumigatus* spores are protected by a layer of the immunological insert polymer, thus enabling it to evade most immune receptors (Garcia-Rubio et al., 2020). However, the AFM particles prepared by grinding the *A. fumigatus* mycelium do not have this type of protective layer. *A. fumigatus* can induce the release of pro-inflammatory cytokines in a TLR2- and TLR4-dependent manner in macrophages (Meier et al., 2003). Similarly, neutrophils can detect *A. fumigatus* via TLR2 and TLR4 receptors in humans (Braedel et al., 2004). No significant stimulation of TLR2 or TLR4 by AFM or AFS was observed using HEK293 reporter cells, suggesting that the doses used here may either be too low or that the pre-treatment of the particles have altered the three-dimensional structure, thus preventing binding to these receptors. In neutrophils and the dHL-60 neutrophil-like cell models, other receptors are responsible for detection of beta-glucans in the *A. fumigatus* mycelium (Kennedy et al., 2007; Saegusa et al., 2009; Werner et al., 2009), which may drive the release of the pro-inflammatory markers.

Microplastics, consistent with other environmental particulate contaminants, elicit robust inflammatory reactions and immune dysfunction across multiple biological scales, from cellular to systemic responses in human populations. Such states are characterized by elevated secretion of pro-inflammatory mediators, such as cytokines (e.g., IL-6 and TNF-α) and chemokines (IL-8), and enhanced activation of immune effector cells, particularly tissue-resident macrophages and circulating neutrophils (Pulvirenti et al., 2022; Tomonaga et al., 2024). Chronic inflammation is a well-established contributor to endothelial dysfunction, atherosclerosis, and the progression of many other chronic diseases (Abbas et al., 2025).

In ambient air and occupational settings, dust exposure generally includes heterogeneous mixtures of diverse particle populations varying in sizes, distribution, morphology, and origins. Although comprehensive characterization of these complex exposure matrices remains incomplete in the scientific literature, mounting evidence suggests that contemporary particulate dust pollution includes microplastics, now recognized as contaminants of emerging concern, alongside fungal bioaerosols with well-characterized immunogenic, allergenic, and toxinogenic properties (Vishwakarma et al., 2023; Yakovenko et al., 2025; Yan et al., 2016).

As the first study of its kind, we investigated the immune-modulating effects of HDPE microplastics and fungal particles in neutrophil-like cells. We hypothesized that mixtures of microplastics and fungal particles, likely representing co-occurring components of outdoor, indoor, and occupational dust, would elicit stronger pro-inflammatory responses than microplastics alone. In fact, particle mixtures consisting of HDPE + AFM (fungal mycelial fragments) induced significantly higher levels of IL-6 and IL-8 than HDPE particles alone. This is not, however, the case with HDPE + AFS (fungal spores) or fungal spores that induced the release of only IL-8. Surprisingly, we observed a decreasing level of IL-6 when the doses of HDPE particles increased, even in combination with fungal particles. These findings are in line with the results reported by Boynton et al. (2000), who also exposed monocyte-derived macrophages to HDPE and reported the release of IL-6. In our study, there is a clear negative trend with measured levels of IL-6 and all treatments containing increasing doses of HDPE. This observation can be attributed to potential loss of secreted IL-6 by adsorption to the test materials, particularly to HDPE, as previously reported for other test materials (Kocbach et al., 2008). Similar findings for IL-6 and TNF-α were reported with PBMCs exposed to PVC and ABS (Han et al., 2020), but not discussed. The cause of such a response remains unclear, and the relationship between increasing microplastic levels and decreasing IL-6 levels, also known as pleiotropic cytokine, cannot be interpreted as a simple cause-and-effect inflammatory response. Instead, it may be a multifaceted reaction influenced by a delicate interplay between various factors of the exposure systems and the microplastic constituents. Although the pro-inflammatory potential of microplastics is commonly reported as a significant concern, evidence also suggests that they can attenuate immune function, especially adaptive immune responses, under specific circumstances (Hua and Wang, 2022). The potential of microplastics to dysregulate the immune system, leaving us more vulnerable to infections and other diseases, represents an insidious threat to human health. As an example, Gupta et al. (2021) showed that increased exposure to particulate matter is associated with increased lethality of COVID-19. Another critical cofounder of this relationship is the increased opportunistic infections such as mucormycosis (Azhar et al., 2022).

Fungal fragments of mycelial origin induced the secretion of the highest levels of IL-6 and IL-8. These results are in line with data reported by Øya et al. (2019) with bronchial epithelial cells (BEAS-2B) and macrophages. *A*. *fumigatus* is a well-characterized model of mold pathogen with pro-inflammatory properties that differ significantly whether the mold is at the mycelia or spore stage. Treatment with the fungal fragments showed the higher levels of measured pro-inflammatory cytokines than fungal spores. This finding corroborates with previous studies reviewed by Pulvirenti et al. (2022).

Another interesting observation is that fungal spores alone induced the release of IL-6 but not IL-8. When co-exposed with HDPE, the opposite response, with the release of IL-8 but not IL-6, was found. These results can be explained by the fact that fungal spores by themselves elicit weak or marginal IL-6 responses and little to no IL-8 responses (Øya et al., 2019), while plastic particles, as revealed for polystyrene microplastics, tend to induce IL-8 production (Mattioda et al., 2023). It is likely that the fungal spores alone trigger a pyrogenic-like response with IL-6 release, whereas in the presence of microplastics IL-8 release is induced, a response that is often characterized as particle effects (Dinarello, 1999).

The inflammatory reactions observed in the present study cannot be used for risk assessment as the neutrophil-like cells used here were differentiated from HL-60 cells of cancerous origin and therefore do not represent a healthy biological system. The HEK293-TLR reporter cells represent an affordable TLR-specific reporter model that are relatively cheap and effective for studying receptor- and pathway-specific responses of the innate immune system *in vitro*. The data generated here can help cover, at mechanistic levels, some of the knowledge gaps of hazards associated with exposure to environmentally relevant microplastics and particularly HDPE MPs. It is, however, important to note that the degree of sensitivity of the HEK293 reporter cell model may be less than that of immune cells responsible for innate immune responses (for example, neutrophils) to pathogen-associated microbial patterns.

## 5 Conclusion

To the best of our knowledge, this is the first study to explore the potential immunotoxic effects associated with microplastic particles in mixture with fungal spores or fungal fragments. Although no cytotoxic effects were observed at the tested doses in the neutrophil-like cell model, pro-inflammatory responses characterized by differential release of IL-6 and IL-8 were found when microplastics were mixed with fungal fragments or spores. Our findings suggest that microbial contaminants, here fungal particles, seem to be the main drivers of the immune responses triggered by combined exposure to HDPE microplastics and fungal particles. In particular, fungal mycelial fragments alone or in mixtures were more potent with significant release of both IL-6 and IL-8 compared to fungal spores that induced only significant release of IL-8. Of note, fungal spores are commonly monitored in risk assessments. Altogether, no or limited effects could be found after exposure to the “pristine” HDPE particles, but exposure to complex mixtures of HDPE and fungal fragments induced significant release of pro-inflammatory markers from the neutrophil-like cells.

## Data Availability

The original contributions presented in the study are included in the article/Supplementary Material; further inquiries can be directed to the corresponding author.
