# Supplementary material for: Microplastics amplify the pro-inflammatory response to fungal mycelial fragments and spores in neutrophil-like cells
Source: Front Toxicol. 2026 Feb 10;8:1718466. doi: 10.3389/ftox.2026.1718466 (PMC12928607; doi:10.3389/ftox.2026.1718466)
Supplement: Supplementary file 1 [file DataSheet1.docx]

**Supplementary Materials**

**Microplastics amplify the pro-inflammatory response to fungal mycelial fragments and spores in neutrophil-like cells**

Anani K Afanou^1*^, Andreas Solberg Sagen^1, 2^, Francesco Barbero^3^, Ilaria Zanoni^4^, Anna Costa^4^; Øyvind P Haugen^1^, Shan Zienolddiny-Narui^1^

^1^ STAMI, National Institute of Occupational Health, Gydas Vei 8, 0363 Oslo, Norway

^2^ UIO, Institute of oral Biology, Sognsveien 10, Postboks 1052, Blindern Oslo, Norway

^3^ UNITO, Department of Chemistry, University of Torino, Torino, Italy

^4^ CNR-ISSMC, Institute of Science, Technology and Sustainability for Ceramics, National Research Council of Italy, Via Granarolo, 64, 48018, Faenza, Italy

*Corresponding author: [anani.afanou@stami.no](mailto:anani.afanou@stami.no)

# **Materials and methods**

## ***Toll-like receptor activation with inverted insert system***

One major issue with buoyant particles in submerged in vitro system is the risk floating leading to reduced or no interaction between the test particles and the cells. With HEK293 reporter cells, we investigated the NFkB induced response through TLR2 and TLR4 activation and the secretion of alkaline phosphatase (SEAP) with HDPE particles in inverted insert systems. In fact, TLR2 (Invivogen #hkb-htlr2) and TLR4 (Invivogen #hkb-htlr4) HEK-Blue reporter cells were grown in Dulbecco's modified Eagle's medium (DMEM; Gibco #31966) supplemented with 10% heat-inactivated endotoxin-free fetal bovine serum (FBS; Biowest #S1860), 100 U/mL penicillin (Biowest #L0022), 100 µg/mL streptomycin (Biowest #L0022), 100 µg/mL Normocin (Invivogen #ant-zn) and 1× HEK Selection-Blue (Invivogen #hb-sel).

HEK-Blue Null parental, hTLR2 and hTLR4 cells (1.76x10^5^ cells in 150µL media) were seeded on inverted 12-well inserts (Falcon cat. #353181) in technical triplicates and incubated at 37oC with 5% CO_2_ for 24h. The inserts with cells were thereafter transferred to 12 well plates containing 900ml cell culture media mixed with test materials (endotoxin free water as negative control, HDPE at 100µg/mL, LTA and LPS at 100ng/mL as positive controls for TLR2 and TLR4, respectively). Following 24 hours exposure, 20µL of the supernatant was mixed with 180µL freshly prepared Quanti Blue solution (Invivogen #rep-qbs) in a new 96-well plate. The plate was incubated for 3 hours at 37 in a 5% CO_2_ saturated atmosphere, and the absorbance was measured at 649 nm in a microplate reader (Agilent Technologies, BioTek Synergy Neo2, Santa Clara, USA). The fold change was calculated as ratio between the test treatments and the negative control. Data reported here were based on two independent experiments.

## ***Cytotoxicity by lactate dehydrogenase with HEK reporter cells in inverted insert systems***

Cytotoxic effects of the treatments were assessed using the CyQUANT™ LDH Cytotoxicity Assay Kit (Invitrogen, USA), which measures the levels of extracellular lactate dehydrogenase (LDH) in the cell culture medium. Following the test material treatments as described above, 50µl of the supernatant were transferred to new 96- well plate and the cells on the basal side of the insert were exposed in 30min to lysis buffer (50µL) for maximal LDH release. 50µL of supernatant were then transferred to the 96-well plate and completed to LDH reagent mix as recommended by the manufacturer. The absorbance was measured, after 30min at 490 nm and 680 nm using a Synergy Neo2 multi-mode reader (BioTek Instruments, USA). Cytotoxicity was expressed as a percentage of background adjusted LDH release from the test material treated cells by the maximum LDH release determined from cells lysed with the assay-provided lysis buffer.

# **Results**

## ***Toll-like receptor activation by HEK293 reporter cell in inverted inserts***

Figure S1 shows the fold change in SEAP activity after 24 hours exposure of reporter cells to HDPE (100 µg/mL) and LTA and LPS (100 ng/mL). The fold change was calculated as ratio of the absorbance of test materials by the average of negative controls. Following 24 hours exposure, none of the receptors (TLR2 or TLR4) were activated by HDPE. However, the positive controls for TLR2 and TLR4 induced significant activation of the respective TLRs.


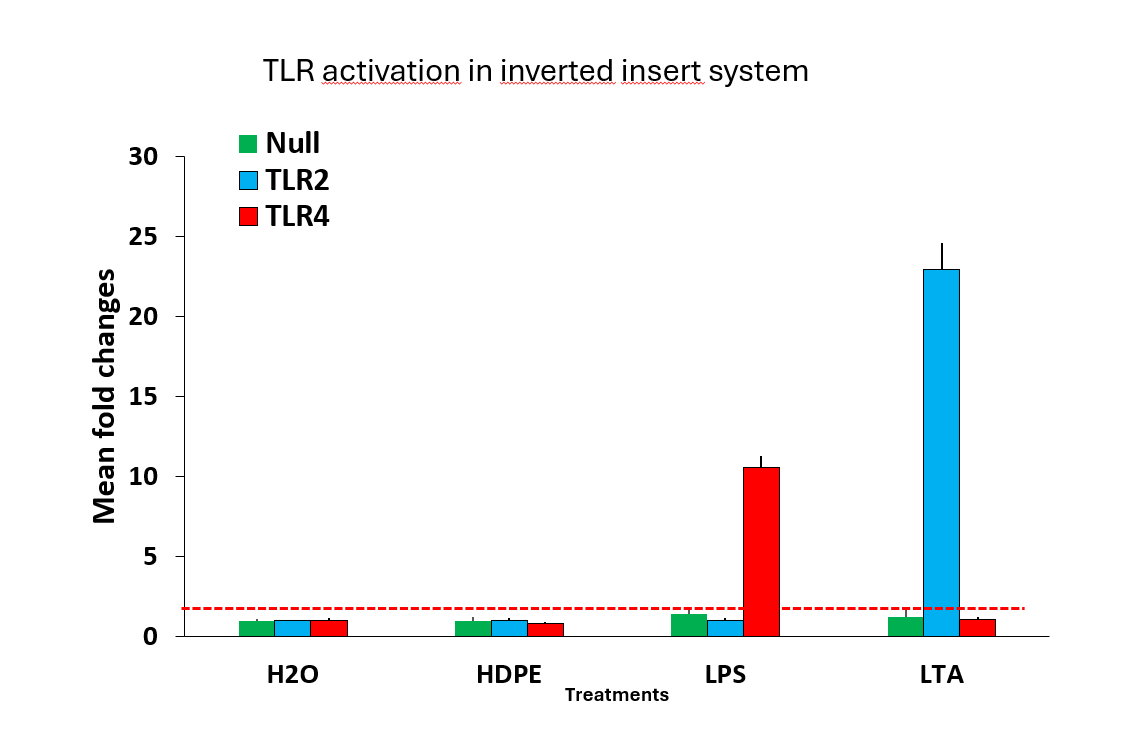


*Figure S1: Bar plots of the fold changes from HEK293 TLR reporter cells under inverted insert transwell system. Test materials include HDPE (100µg/mL), H_2_O as negative control and positive controls (LPS (100ng/mL) for TLR4 and LTA (100ng/mL) for TLR2). The red stippled line indicates fold change level 2 as the activation threshold. Data reported as mean with standard deviation of two experiments.*

## Cytotoxicity by LDH

The cytotoxic effects of HDPE and positive controls in the insert transwell system are summarized as bar plot in Figure S2.

*Figure S2: Bar plots of the cytotoxicity in HEK293 TLR reporter cells under inverted insert transwell system. Test materials include HDPE (100µg/mL), H_2_O as negative control and positive controls (LPS (100ng/mL) for TLR4 and LTA (100ng/mL) for TLR2). Data reported as mean with standard error of two experiments.*
